# Effects of stereotactic aspiration on brainstem hemorrhage in a case series

**DOI:** 10.3389/fsurg.2022.945905

**Published:** 2022-08-19

**Authors:** Lei Du, Ji-Wei Wang, Cong-Hui Li, Bu-Lang Gao

**Affiliations:** Department of Neurosurgery, The First Hospital of Hebei Medical University, Shijiazhuang, China

**Keywords:** brainstem, hemorrhage, stereotactic aspiration, surgery, complications

## Abstract

**Purpose:**

Brainstem hemorrhage is usually treated conservatively with medication and has high mortality and morbidity rates. Stereotactic aspiration can directly and microinvasively draw out the hemorrhage within a narrow space in the brainstem, thus promoting quick recovery and potentially saving the life of the patient. This study was conducted to investigate the effect of stereotactic aspiration on patients with brainstem hemorrhage in a case series.

**Materials and methods:**

A total of 42 patients with brainstem hemorrhage were enrolled for stereotactic aspiration of the brain hemorrhage, and another 30 patients with brainstem hemorrhage were enrolled for conservative treatment. The clinical and imaging data were analyzed and compared.

**Results:**

Stereotactic aspiration was successful in all patients (100%), with immediate elimination of hematoma in the brainstem. In five patients with the hemorrhage extending to the fourth ventricle (*n* = 1) and basal ganglia (*n* = 4), the hemorrhage was eliminated, resulting in good outcomes. However, four patients died of multiple organ failure after aspiration, resulting in a mortality rate of 9.5%. One week after surgery, the Glasgow Coma Scale (GCS) score ranged from 3 to 11 (mean 5.9 ± 2.3). At 1-month follow-up, 4 patients died, and 36 patients survived, with the GCS score ranging between 3 and 15 (mean 8.6 ± 2.1), which was significantly (*P* < 0.01) higher than that before surgery. The Modified Rankin Scale (mRS) score was 5 before treatment, 5 (4.4, 6) at 1 week after surgery, and 5 (4, 6) at 1 month. In the conservative group, 16 (53.3%) patients died during hospitalization. The GCS score was 0–6 (mean 2.3 ± 1.1), which was significantly (*P* < 0.05) worse than at admission or of that in the aspiration group at 1 month. The mRS score at 1 month was 6 (5, 6), which was significantly (*P* < 0.05) worse than that in the aspiration group.

**Conclusion:**

Stereotactic aspiration for brainstem hemorrhage as an approach of microinvasiveness may be effective in evacuating brainstem hemorrhage and may promote quick recovery of the patient, resulting in better clinical outcomes.

## Introduction

Hypertensive brainstem hemorrhage denotes a catastrophic event with an extremely poor outcome (1, 2). The rate of incidence of primary brainstem hemorrhage is 2%–4% in 100,000 people every year, accounting for up to 10% of the cases of intracerebral hemorrhage, and the most common type is pontine hemorrhage, with a prevalence of 6%–7% and a mortality rate ranging from 40% to 50%. The prognosis for brainstem hemorrhage is very poor with a high mortality rate ranging from 30% to 90%, while the rate of favorable outcomes for patients receiving conservative therapy is very low, ranging from 15.3% to 78% (2–4). Brainstem hemorrhage usually leads to serious conscious disturbance, motor damage, and respiratory damage. Currently, it is being debated whether to manage brainstem hemorrhage with conservative or surgical approaches, with some researchers suggesting conservative therapy (1, 5, 6) but others advocating for surgical treatment (1, 7–9), because recent advances in microsurgery and neuroimaging techniques, intraprocedural neurophysiological monitoring, and neuronavigation have allowed for improvements in the outcomes of patients with primary brainstem hemorrhage. However, the surgical efficacy of brainstem hemorrhage remains debatable.

Aspiration of intracerebral hematoma with stereotactic surgery has been attempted for a long time, and Backlund and von Holst were the first to perform a successful aspiration of intracerebral hematoma at the acute stage in 1978 (10, 11), and since then, computed tomography (CT)-guided stereotactic surgery has been established for intracerebral hematoma, especially large hematomas at the acute stage, and tumors for biopsy. The aim of this procedure is to improve neurological functional recovery in patients with moderate and severe neurological deficits. Stereotactic aspiration has mostly been applied for intracranial hematomas, with varying effects (11–16), and a few studies have been performed to investigate the effect of stereotactic aspiration on brainstem hemorrhage. Beatty and Zervas used stereotactic aspiration to treat a brainstem hematoma in a patient, resulting in good recovery (17). In investigating the effect of CT-guided stereotactic aspiration of hypertensive pontine hemorrhage in 20 patients compared with conservative treatment for another 25 patients, Shitamichi et al. (18) achieved good outcomes in nine patients, fair in four, and poor in the remaining seven, with no death or deterioration after surgery in the aspiration group. For patients with a brainstem hematoma <5 ml, a good clinical outcome was achieved in both the conservative and the aspiration groups. For patients with a hematoma ranging from 5 to 10 ml, a good prognosis was achieved in the aspiration group but a poor one in the conservative group because there were six deaths. For patients with a hemorrhage greater than 10 ml, a poor outcome was observed in the aspiration group, but a miserable outcome (including 11 deaths) was observed in the conservative group (18). These reports demonstrated that stereotactic aspiration for brainstem hemorrhage had better outcomes compared with conservative therapy. We hypothesized that stereotactic aspiration could lead to good effects on patients with brainstem hemorrhage, and therefore, in this study, we sought to investigate the effects of this procedure on such patients.

## Methods

This retrospective case-series study performed at a single center was approved by the ethics committee of our hospital, with all patients giving their signed informed consent. Between October 2018 and October 2019, consecutive patients with brainstem hemorrhage were enrolled in the study for stereotactic aspiration of the intracranial hemorrhage at a tertiary hospital (First Hospital) of Hebei Medical University. The inclusion criteria were as follows: patients with brainstem hemorrhage, decreased breathing or apnea with mechanical ventilation support, marked conscious dysfunction (Glasgow Coma Scale score or GCS < 12), volume of hemorrhage >3 ml deviating toward one side or the dorsal side, and hemorrhage within 72 h. The exclusion criteria were as follows: slight conscious dysfunction (GCS ≥ 12), slight neurological damage (muscle strength ≥ 3), bilateral pupil dilation, unstable circulation (systolic/diastolic pressure less than 12/8 kPa, respectively), head trauma, coagulopathy, cerebrovascular anomaly, brainstem failure, and hemorrhage beyond 72 h.

A CT scan was performed in all patients with a slice thickness of 5 mm, and the size and volume of the hematoma were measured according to the formula of *A* × *B* × *C*/2, with *A* standing for the greatest diameter of the hematoma on the axial plane of CT scanning, *B* for the diameter of the hematoma perpendicular to *A* on the same plane, and *C* for the total length on the vertical plane (2, 15). Preoperative preparation included endotracheal intubation, gastrointestinal decompression, and preparation of the head. Medications were administered for maintenance of normotension. Under local anesthesia conditions, the Anke stereotactic head frame (Anke Operation Plan System, Anke, Shenzhen, China) was installed. A head CT scan was performed. After the head was correctly positioned in the apparatus, the *X*-, *Y*-, and *Z*-axes coordinates were calculated on the CT scan, and the center of the hematoma was selected as the targeted point. Under general anesthesia, a hole was drilled in the skull below the transverse sinus to avoid the venous sinus, and the dura was opened in the shape of a “cross.” Based on the hematoma morphology, the hematoma long axis was chosen as the puncture route of a hematoma puncture needle. It was relatively safe to pass the puncture needle through the cerebellum–dorsal brainstem or the cerebellum–fourth ventricle–dorsal brainstem. A hematoma puncture needle was slowly sent to the hematoma center, and after the needle core was withdrawn, a 5-ml syringe was used to slowly aspirate the hematoma. During the aspiration process, a small amount of warm physiological saline was used to dilute the hemorrhage. First, a 2-ml hemorrhage was aspirated, and then a 2-ml warm physiological saline was injected into the hematoma. The aspiration was repeated with reduced amounts of both aspirated hemorrhage and injected physiological saline until most of the blood was aspirated. Then, the syringe was withdrawn, and a tube was put in place for drainage. Before surgical aspiration, the patient was evaluated with the GCS score, and after the surgery and at follow-up, the GCS and Modified Rankin Scale (mRS) score were used to assess the patient.

## Statistical analysis

Statistical analysis was performed by using the SPSS software (IBM, Chicago, IL, USA). Measurement data were presented as mean ± standard deviation and tested by using the paired *t*-test, while enumeration data were presented as percentage and tested with the *χ*^2^ test. The significant level was set at *P* < 0.05.

## Results

Between October 2018 and October 2019, 42 patients with brainstem hemorrhage were enrolled to undergo stereotactic aspiration treatment (aspiration group), of which 36 were men and 6 were women with an age range of 29–73 years (mean 47.4 ± 10.0, Table 1). The duration from disease onset to aspiration was within 6 h in five cases, 6–24 h in 35 cases, and 24–72 h in only the remaining two. The hemorrhagic location was in the pons in 23 (54.8%) patients; pons and midbrain in 14 (33.3%); pons, midbrain, and basal ganglia in 3 (7.1%); pons and basal ganglia in 1 (2.4%); and midbrain and left thalamus in 1 (2.4%). The hemorrhagic volume ranged 5–13 ml (mean 9 ± 4 ml), and the GCS score ranged 3–7 (mean 3.8 ± 1.3).

**Table 1:** Demography and treatment in the stereotactic aspiration and conservative groups.

Variables	Stereotactic aspiration	Conservative treatment	*P*
No.	42	30	
M/F	36/6	22/8	0.56
Age (mean, year)	29–73 (47.4 ± 10.0)	45–60 (48.0 ± 4.5)	0.8
Duration of disease (h)	6–72	5–72	0.23
Hemorrhage location (%)			0.34
Pons	23 (54.8%)	14 (46.7%)	
Pons and midbrain	14 (33.3%)	8 (26.7%)	
Pons, midbrain, and basal ganglia	3 (7.1%)	5 (16.7%)	
Pons and basal ganglia	1 (2.4%)	3 (10%)	
Midbrain and left thalamus	1 (2.4%)	0	
Hemorrhage volume (ml)	5–13 (9.0 ± 4)	5–12 (8.6 ± 3.5)	0.9
Death	11.8 ± 4.2	10.9 ± 4.8	0.83
Alive	8.2 ± 3.6*	5.8 ± 3.2*	0.03
Pretreatment GCS	3–7 (3.8 ± 1.3)	3–7 (3.6 ± 1.5)	0.6
Posttreatment GCS (1 m)	3–15 (8.6 ± 2.1)	0–6 (2.3 ± 1.1)	<0.001
Death (*n*, %)	6 (14.3%)	16 (53.3%)	
Pretreatment mRS	5	5	
1 week after surgery	5 (4.4, 5)	6 (5, 6)	0.04
1-month follow-up	5 (4, 6)	6 (5, 6)	<0.05

GCS, Glasgow Coma Scale; mRS, modified Rankin Scale.

* A significant (*P* < 0.05) difference in the hemorrhage volume when comparing patients who survived and those who died.

During the same period, 30 patients (22 men and 8 women) with brainstem hemorrhage and aged 45–60 years (mean 48.0 ± 4.5) were enrolled in the conservative control group (Table 1). The duration from disease onset to admission was 5–72 h, and the hemorrhagic volume was 5–12 ml (mean 8.6 ± 3.5). The hemorrhagic location was in the pons in 14 (46.7%) patients; pons and midbrain in 8 (26.7%); pons, midbrain, and basal ganglia in 5 (16.7%); and pons and basal ganglia in 3 (10%). The GCS score ranged 3–7 (mean 3.6 ± 1.5). No significant (*P* > 0.05) difference existed in the sex percentage, age, duration from onset to treatment, hemorrhagic location and volume, and GCS score between the two groups of patients.

**Figure 1:**
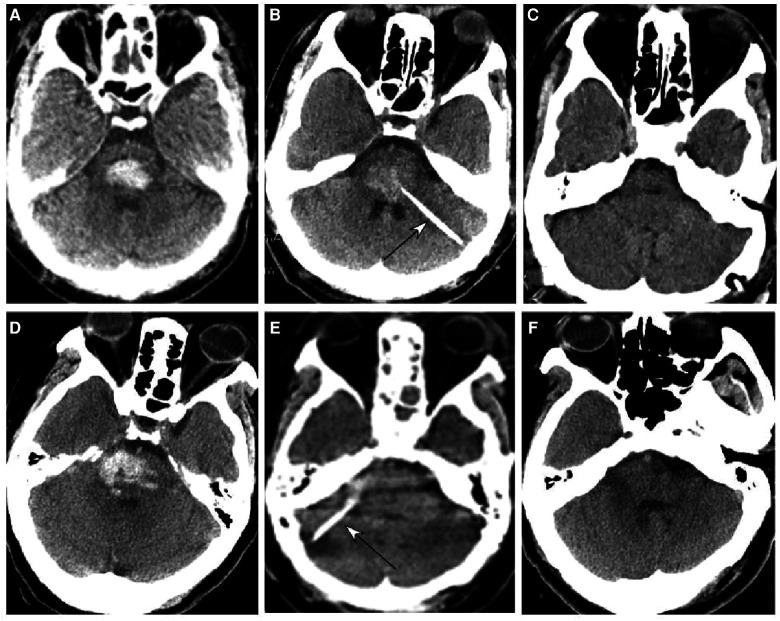
Brainstem hemorrhage treated with stereotactic aspiration. (**A**–**C**) A 59-year-old man had a sudden onset of unconsciousness 2 h before presentation. (**A**) Computed tomography (CT) revealed brainstem hemorrhage. Before the operation, he was on mechanical ventilation and in a moderate state of coma with a Glasgow Coma Scale (GCS) score of 5 (E1VTM4). (**B**) Stereotactic aspiration was performed (arrow). The arrow indicates the aspiration needle. On the third day following surgery, he regained consciousness and could get down from the bed with a GCS score of 14 (E4V4M6) and a Modified Rankin Scale score of 3. (**C**) Five months after surgery, the patient could walk and live without assistance. (**D**–**F**) A 65-year-old woman had a sudden loss of consciousness for 1 day. (**D**,**E**) CT imaging showed brainstem hemorrhage. Before surgery, she was in a moderate state of coma with GCS 6 (E2VTM4) and on mechanical ventilation (**D**,**E**). (**F**) Stereotactic aspiration was performed (arrow). After the surgery, the patient became conscious and could move her hands with a GCS score of 10 (E4VTM 6) and a modified Rankin Scale score of 4.

**Figure 2:**
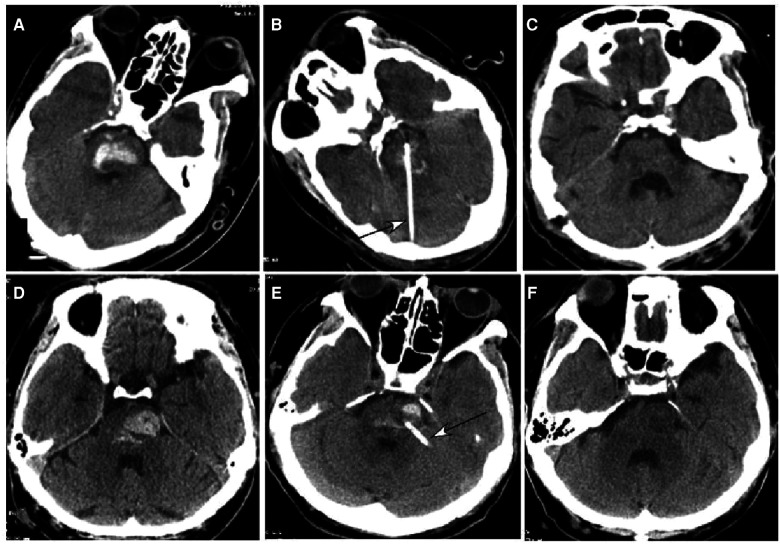
Brainstem hemorrhage. (**A**–**C**) A 52-year-old man had a sudden loss of consciousness 5 h before presentation. Before surgery, he was in a coma with a Glasgow Coma Scale (GCS) score of 5 (E1VTM4) supported with mechanical ventilation. (**A**) Computed tomography (CT) revealed brainstem hemorrhage. (**B**) Stereotactic aspiration was performed (arrow). (**C**) After surgical aspiration, the hematoma in the brainstem disappeared, and he became conscious with a GCS score of 10 (E4VTM6) and a Modified Rankin Scale score of 4. Twenty days later, he could move his hands. (**D**–**F**) A 46-year-old man had a sudden onset of unconsciousness with nausea and vomiting for three hours before presentation. (**D**) CT imaging demonstrated brainstem hemorrhage. Before surgery, he was in a coma with a GCS score of 5 (E1VTM4) and mechanical ventilation. **(E**) Stereotactic aspiration was performed (arrow). (**F**) Three weeks after the surgery, the hemorrhage in the brainstem disappeared, and he regained his consciousness with a GCS score of 10 (E4VTM6) and a Modified Rankin Scale score of 4.

**Figure 3:**
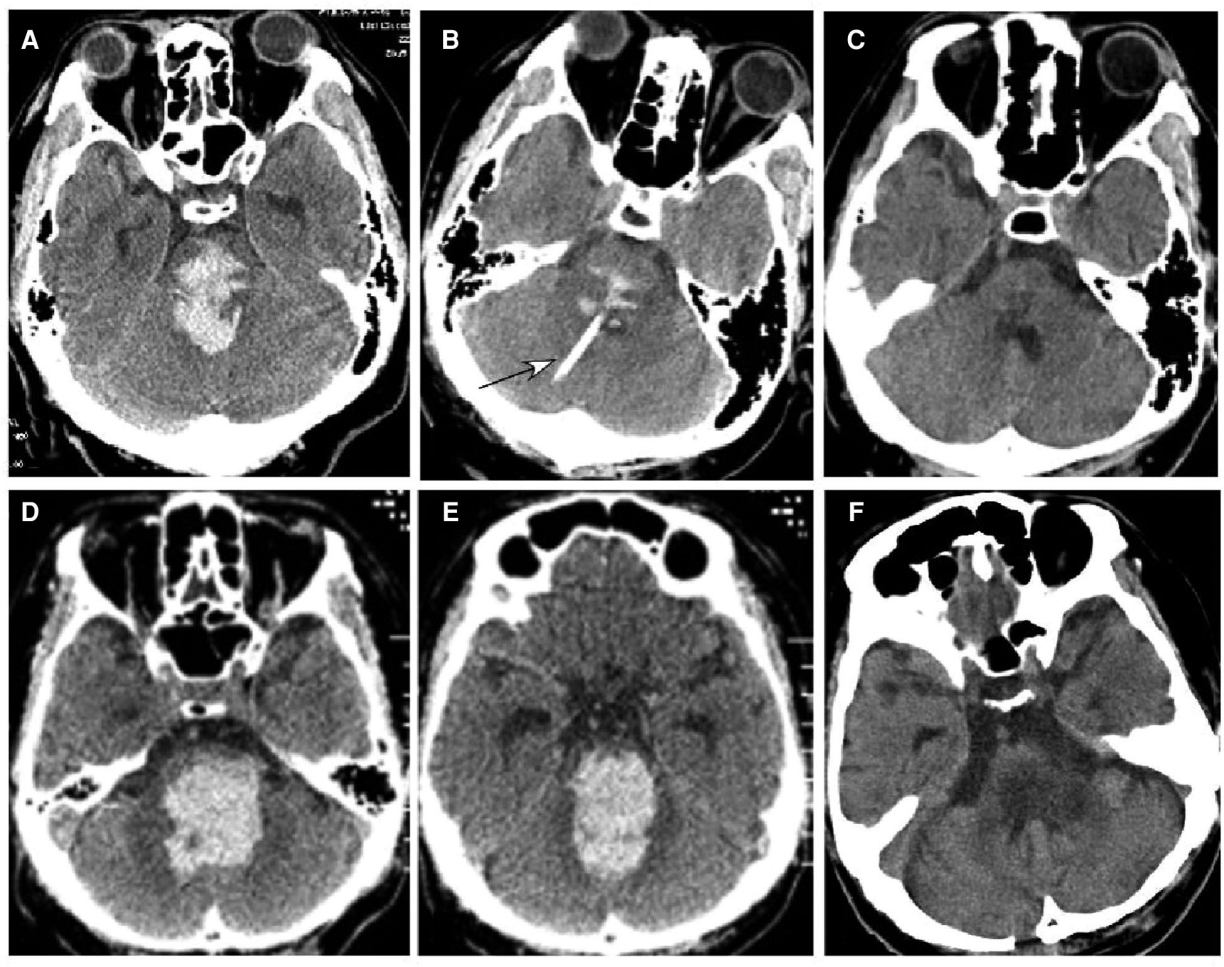
Brainstem hemorrhage. (**A**–**C**) A 48-year-old man had a sudden loss of consciousness 6 h before presentation. He was in a coma with a Glasgow Coma Scale (GCS) score of 3 (E1VTM2) but no spontaneous breathing before surgery. Computed tomography (CT) revealed brainstem hemorrhage (**A**). Stereotactic aspiration was performed (**B**, arrow). One month after surgery (**C**), the hemorrhage disappeared, and he regained his spontaneous breathing and could open his eyes with the GCS of E4TM5. (D–**F**) A 48-year-old man had a sudden onset of unconsciousness 6 h before being transferred to our hospital. CT scan showed severe hemorrhage in the brainstem and the fourth lateral ventricle (**D**,**E**), and he was in a coma with a GCS score of 3 (E1VTM2) but no spontaneous breathing. Preoperative estimation was that he might not survive, but his family was strongly in favor of surgical treatment. One week after the stereotactic aspiration (**F**), he could open his eyes, and on the ninth day, the hemorrhage disappeared completely.

**Figure 4:**
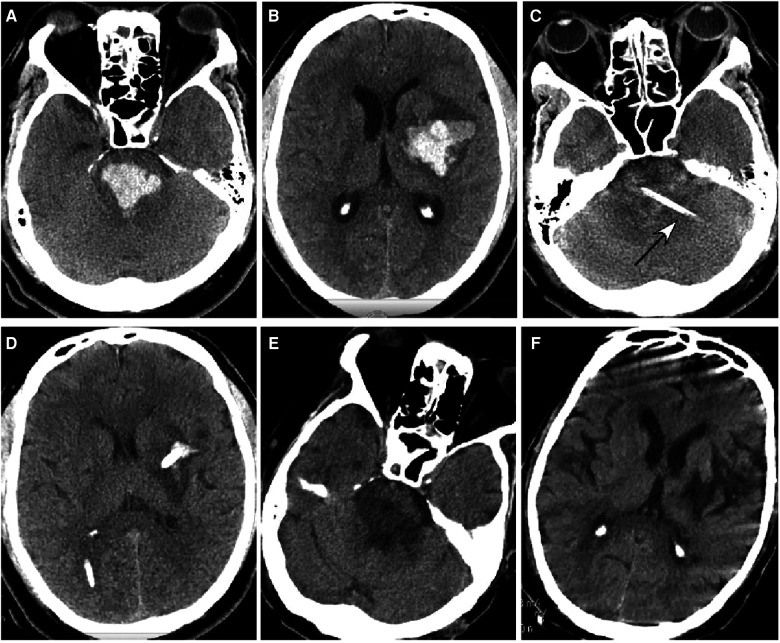
Brainstem hemorrhage. (**A**,**B**) A 45-year-old man had a sudden loss of consciousness 1 h before presentation. Head CT scan (**A**,**B**) showed severe hemorrhage in the brainstem, thalamus, and basal ganglia. The patient was in a coma with a Glasgow Coma Scale (GCS) score of 3 (E1VTM2). (**C**,**D**) An ultra-early stereotactic aspiration was performed (arrow). (**E**,**F**) Three months after surgery, the patient had a GCS score of 6 (E3VTM4) and was in active rehabilitation.

**Figure 5:**
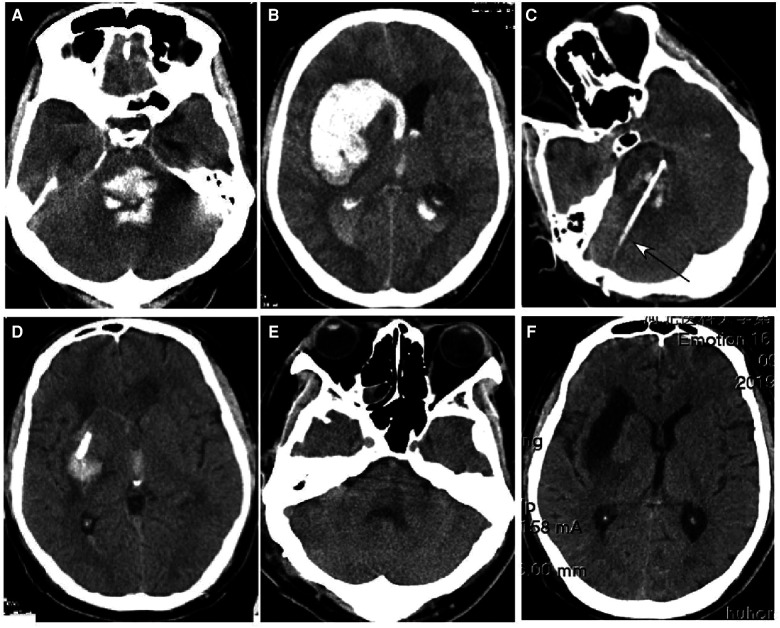
A 39-year-old man had a sudden onset of unconsciousness 3 h before presentation. (**A**,**B**) A head CT scan showed severe hemorrhage in the brainstem (**A**), basal ganglia, and lateral ventricle (**B**). The patient was in a coma with no spontaneous breathing and with a Glasgow Coma Scale (GCS) score of 3 (E1VTM2). (**C**,**D**) An ultra-early stereotactic aspiration was performed (arrow). After surgery, the hemorrhage greatly reduced, and the patient regained his spontaneous breathing with a GCS score of 4 (E1VTM3). (**E**,**F**) One month later, the hemorrhage had completely disappeared.

The stereotactic aspiration procedure was performed in the stereotactic aspiration group and lasted about half an hour, and all patients had successful aspiration (100%) with no surgery-related deaths (Table 1, Figures 1–5). An immediate CT head scan revealed no presence of hematoma in the brainstem (Figures 2–4). In five patients with the brainstem hemorrhage extending to the fourth ventricle (*n* = 1) and basal ganglia (*n* = 4), immediate stereotactic aspiration eliminated the hemorrhage volume, resulting in good outcomes (Figures 3–5). Four patients died of multiple organ failure after aspiration, resulting in a mortality rate of 9.5%. One week after surgery, the GCS score ranged 3–11 (mean 5.9 ± 2.3). At 1-month follow-up, four patients died, and 36 patients survived, with the GCS score ranging between 3 and 15 (mean 8.6 ± 2.1), which was significantly (*P* < 0.01) greater than that before surgery. The mRS score was 5 before treatment, 5 (4.4, 5) at 1 week after surgery, and 5 (4, 6) at 1 month. In a comparison of the hemorrhagic volumes between patients who survived and those who died, a significant (*P* < 0.05) difference existed in the hemorrhagic volume (Table 1), with this volume being significantly greater in patients who died than in those who survived (11.8 ± 4.2 vs. 8.2 ± 3.6 ml).

In the conservative group (Table 1), 16 (53.3%) patients died during hospitalization. The GCS score was 0–6 (mean 2.3 ± 1.1), which was significantly (*P* < 0.05) worse than at admission or of that in the aspiration group at 1 month. The mRS score at 1 month was 6 (5, 6), which was significantly (*P* < 0.05) worse than that in the aspiration group. The hemorrhagic volume in patients who died was significantly greater than that in patients who survived (10.9 ± 4.8 vs. 5.8 ± 3.2 ml).

## Discussion

This study investigated the clinical outcomes of patients with brainstem hemorrhage treated with stereotactic aspiration or conservatively, and it was found that stereotactic aspiration could significantly improve the clinical outcomes with a significantly decreased death rate. The advantages of stereotactic aspiration for brainstem hemorrhage over conventional open surgery for hematoma elimination lie in the following facts: short operation time, rapid and early evacuation of the hematoma, which is especially valuable for hemorrhage in the brainstem with limited space, minimal surgical invasion with a lower surgery-associated morbidity, and microinvasive surgery to enable treatment in elderly patients harboring comorbidities (19).

In the study reporting the experiences in managing brainstem hematomas, Rohde et al. (20) presented 15 comatose patients with brainstem hemorrhage who were managed medically with (*n* = 4) or without (*n* = 11) additional external ventricular drainage because of ventricular dilatation in acute occlusive hydrocephalus, and all of these patients died within days of hemorrhage. After analyzing 32 patients with brainstem hemorrhage treated conservatively, Komiyama et al. (5) reported the death of 11 patients (34.4%) who were comatose at the initial presentations, minimal neurological deficits in 10 patients (31.3%) who survived and resumed normal activities, and 11 patients (34.4%) rendered bedridden, necessitating full living support. In a study of the clinical and neuroradiological predictors of mortality in 32 patients with primary pontine hemorrhage treated medically, Balci et al. (21) found that 18 patients (56%) died and 14 patients (44%) survived, with significant predictors for mortality as coma at admission (*P* = 0.001), intraventricular extension (*P* = 0.019), extrapontine extension (*P* = 0.001), hydrocephalus (*P* = 0.024), necessity of mechanical ventilation (*P* = 0.007), and massive and bilateral tegmental localization (*P* = 0.006). It seems that most comatose patients with brainstem hemorrhage will die if treated conservatively. In our study, all patients were comatose, but stereotactic aspiration saved the lives of most patients in the stereotactic aspiration group. No surgery-related deaths occurred; however, four patients (9.5%) died of multiple organ failure after aspiration. In the conservative group, the mortality rate was as high as 53.3% (16 out of 30 patients).

The time to treatment is an extremely important factor for prognosis because hematoma elimination as early as possible can relieve not only the mass effect but also secondary brainstem injury induced by surrounding vasogenic edema. Some authors have suggested better outcomes if managed with surgery within 6 h after disease onset (1). In the study of surgical management of spontaneous hypertensive brainstem hemorrhage in five patients with a GCS of 3–7 (mean 5) (1), Shrestha et al. reported a better prognosis in patients who were treated with surgical evacuation of the brainstem hematoma within 6 h of onset, including two patients being able to do all daily tasks without any help, one wheelchair-bound patient needing some help to perform routine daily activities, and two bedridden patients at a follow-up of 6 months. These results were probably caused by rapid elimination of the hematoma, which can clear both the mass effect of the hematoma and the secondary injury caused by surrounding vasogenic edema. In the brainstem, one vital structure is the reticular formation, whose damage by hemorrhage can cause immediate and irreversible loss of consciousness (21). Timely elimination of brainstem hematoma can clear not only the mass effect caused by the hematoma but also the secondary effects of brainstem injury induced by surrounding vasogenic edema. In our study, an ultra-early stereotactic aspiration was performed within 6 h after hemorrhage in five patients who had the hemorrhage extending to the lateral ventricle and basal ganglia (Figures 4, 5), which was a severe condition, and this early aspiration could immediately eliminate these harmful effects and prevent worse clinical outcomes.

In a study investigating the effect of the anterior subtemporal surgical approach for 28 patients with severe upper pontine hematomas compared with 34 patients with a large amount of hematoma (>5 ml) treated conservatively, Zhang et al. (22) reported a death rate of 35.7% (*n* = 10), a 28.6% rate of good function (*n* = 8), a 10.7% disability rate (*n* = 3), and a 25% vegetative rate (*n* = 7) in the surgical group but a 73.5% death rate (*n* = 25) and a 14.7% rate of good function (*n* = 5) in the conservative group. This study clearly demonstrated better effects in surgery than in conservative treatment. After investigating the predictors of surgical effects in 45 patients with large (>5 ml) primary pontine hemorrhage and a GCS score <8, Tao et al. (23) reported a 30-day mortality rate of 31.1% (*n* = 14) and a favorable functional recovery rate of 15.6% (*n* = 7) within 3 months, with the patients who died being significantly older and having a lower GCS score, a larger hematoma volume, and rostrocaudal and ventricular extension of the hematoma.

The size of the hematoma and the level of consciousness on admission are associated with high mortalities and worsened dysfunction outcomes (4, 18, 23–25). In a study of CT-guided stereotactic aspiration for pontine hemorrhages (18), Shitamichi et al. reported that patients with a hematoma volume of 5–10 ml had better prognosis than those with >10 ml hematoma volume after hematoma aspiration. After studying survival outcomes in 32 patients with brainstem hemorrhage (5), Komiyama et al. found a good prognosis in patients with a hematoma of less than 2.5 cm. In the study of CT findings and clinical features as markers for patient outcome in primary pontine hemorrhage (25), Wessels et al. reported a high correlation between a poor prognosis (GOS score <4) and hematoma volume >4 ml, ventral hemorrhage, and a necessity for mechanical ventilation. In their study, a significantly better prognosis was achieved in patients with dorsal hematomas <4 ml in volume. In cases with a slighter disturbance of consciousness, stereotactic aspiration could have a favorable functional outcome (9), and a GCS score on admission which was not related to the hematoma volume may have an impact on the surgical outcome although a large hematoma may frequently causes severe disturbance in consciousness (23). In our study, the clinical outcomes did not correlate with the hematoma volume, and even in patients with the brainstem hemorrhage extending to the ventricles and basal ganglia, immediate stereotactic aspiration could save the patient's life, achieving good outcomes. Only if both the hematoma mass effect and the secondary brainstem injury are eliminated immediately can a worse clinical prognosis be prevented. In a study evaluating the effect of CT-guided stereotactic surgery in aspirating hypertensive intracerebral hematoma using the Archimedes screw between 1 and 24 days (mostly within 1 week) (11), Tankawa et al. found that consciousness improvement could be obtained within a short period of time in most of the 46 cases, perifocal edema decreased much earlier than those managed with medications only, and even patients operated on in the chronic stage experienced increased spontaneous activity but decreased hypersomnia. Thus, it seemed that CT-guided stereotactic surgery provided faster recovery.

Brainstem hemorrhage usually dissects or pushes rather than destroys surrounding structures and can expand quickly to the ventricles and basal ganglia, endangering the life of the patient. This effect, combined with the secondary surrounding vasogenic edema, can lead to a coma and disturbance of vegetative functions (disturbance of respiration, hyperthermia, cardiac dysrhythmias, and hypertension) (1). For patients with compression of the pyramidal tract, abducens nerve and oculomotor nuclei, facial nerve loop and nucleus, and reticular formation, timely elimination of the brainstem hemorrhage will improve eye movement and motor function and help regain consciousness by clearing disturbance. The toxic effects resulting from the disintegration of the hematoma can cause chemical injury to the cells if the hematoma is not eliminated quickly. The mass compression effect, secondary edema, and toxic effects will lead to worse clinical outcomes in patients treated conservatively. Elimination of these effects is the mechanism of surgical evacuation and aspiration. Stereotactic aspiration is a microinvasive approach and is suitable for older patients with other comorbidities who cannot tolerate open surgical procedures. External drainage of the lateral ventricle helps decrease the intracranial pressure, further benefiting the patient for recovery. In our study, all patients had an external drainage of the lateral ventricle irrespective of hemorrhage extension. Theoretically, brainstem hemorrhage can quickly lead to a degeneration and necrosis of neurons surrounding the hemorrhage, and evacuation of the hemorrhage will hardly restore the vitality of these neurons. Actually, in our daily practice with stereotactic aspiration combined with lateral ventricular drainage, we have observed that patients with brainstem hemorrhage could show significant clinical improvement 2 or 3 days after stereotactic aspiration, while those treated conservatively had rare clinical improvement and even deterioration of the conditions after liquefication of the hematoma and aggravation of perifocal edema. This is probably caused by the presence of more fiber bundles and fewer neurons in the brainstem, making the brainstem tolerable to hematoma compression, and timely evacuation of the hematoma can eliminate the compression effect and restore the function of the brainstem.

Simple aspiration was initially advocated as a treatment modality in the 1950s but was abandoned because of significant hematoma remnants at surgery or autopsy (19, 26). The two major challenges initially facing aspiration for intracerebral hemorrhage were targeting the hematoma and difficulty in completely aspirating the hematoma due to heterogeneity of the clot component. Within the initial few hours of hemorrhage, the hematoma comprises about 20% liquid blood and 80% denser clot by volume, which makes simple aspiration very difficult (19, 27, 28). With the advent of stereotactic techniques, targeting the lesion is not an issue any longer, and complete elimination of the hematoma has become the aim. Some instruments and drugs were developed to shatter and liquefy the consolidated hematoma besides increasing the volume of aspiration, including an Archimedes screw, which enabled almost complete aspiration of the clot (29). However, until now, stereotactic aspiration has still not been popularized, with most of these practices focusing on hemorrhage in other locations of the brain rather than the brainstem. Currently, the largest series of patients with brainstem hemorrhage treated with stereotactic aspiration was reported by Shitamichi et al., with only 20 patients being treated this way (18). In this series of patients, good outcomes were achieved in nine patients, fair in four, and poor in the remaining seven, with no death or deterioration after aspiration. During the first week following the aspiration procedure, activity recovery was gained in 75% of the patients, motor function improvement in 75%, and ocular motility recovery in 35% of the patients, with no other deterioration following the procedure.

The strength of this study was the use of the stereotactic aspiration procedure to directly and microinvasively draw out the hemorrhage within a small space in the brainstem, which facilitates quick recovery, decreases morbidity and mortality, and improves the quality of life for patients who survived. This study also had some limitations: it was a one-center study, it was retrospective in nature, only Chinese patients enrolled in it, and it had a short follow-up duration. In the future, a prospective, randomized study with the involvement of multiple centers and other ethnicities is necessary for better evaluation of the effect of stereotactic aspiration in patients with brainstem hemorrhage.

In conclusion, stereotactic aspiration for brainstem hemorrhage as an approach of microinvasiveness may be effective in evacuating brainstem hemorrhage and may promote quick recovery of the patient, resulting in better clinical outcomes.

## Data Availability

The datasets presented in this study can be found in online repositories. The names of the repository/repositories and accession number(s) can be found in the article/Supplementary Material.
